# Coconut Water: An Unexpected Source of Urinary Citrate

**DOI:** 10.1155/2018/3061742

**Published:** 2018-11-01

**Authors:** Roshan M. Patel, Pengbo Jiang, John Asplin, Ignacio Granja, Taylor Capretz, Kathryn Osann, Zhamshid Okhunov, Jaime Landman, Ralph V. Clayman

**Affiliations:** ^1^Department of Urology, University of California, Irvine, USA; ^2^Litholink Corporation, Laboratory Corporation of America® Holdings, USA; ^3^Department of Medicine University of California, Irvine, USA

## Abstract

**Purpose:**

Coconut water has long been touted for its medicinal qualities including natural hydration. We sought to determine whether its consumption would induce changes to urinary lithogenic factors beyond changes in urine volume.

**Materials and Methods:**

After Institutional Review Board approval, volunteers with no prior history of nephrolithiasis were recruited. Each participant was randomized initially to either the coconut water or the water phase of the study. Participants kept meticulous food and fluid intake logs during the first phase of the study and were asked to replicate that diet for the second phase. For each phase the participant consumed 2L of either Taste of Nirvana® pure coconut water or tap water daily for four days. Participants were not restricted to consume additional fluid of their choice during their assigned study phase. During days 3 and 4 of each phase the participant collected a 24-hour urine specimen. Coconut water citrate and malate content were measured and were used along with the beverage pH to calculate the total alkali content of the coconut water. Supersaturation levels were calculated using Equil2. Nonparametric paired analysis using the Wilcoxon test was performed for statistical analysis.

**Results:**

There were 4 adult male and 4 adult female participants. Each individual's 24-hour urine collection had a creatinine excretion within 20% of the mean for each subject's four samples corroborating that all samples were collected properly. The two samples from each phase for each individual were averaged. The coconut water itself was also analyzed and it was calculated to have a total alkali content of 13.8 mEq/L. Consumption of coconut water significantly increased urinary citrate (29%, p=0.02), urinary potassium (130%, p=0.01), and urinary chloride (37%, p=0.03), without affecting urine pH (p=0.16) or volume beyond that of tap water (p=1.00).

**Conclusions:**

Coconut water consumption increases urinary potassium, chloride, and citrate in nonstone forming individuals.

## 1. Introduction

The worldwide prevalence of kidney stones has increased dramatically over the past few decades with calcium oxalate nephrolithiasis continuing to be the most common type of urolith in the United States of America. [1, 2] In patients with calcium oxalate nephrolithiasis, hypocitraturia is found in up to 60% of samples on quantitative 24-hour urine chemistry. [3] The mainstay medical treatment in these patients is potassium citrate; however, adherence to this supplement is notoriously poor given the frequency to take the medications (usually three times a day), the number of tablets needed, cost, and side effects. [4] Alternatively, patients are also counseled on dietary modifications and they are encouraged to increase their consumption of fluids high in citrate content (e.g., lemonade, crystal light).

Coconut water is the liquid endosperm of green coconuts (Cocos nucifera L.), which is the most naturally widespread fruit plant on Earth. [5] Known to Hawaiian's as Noelani, meaning “dew from the heavens,” it is rich in electrolytes, vitamins, minerals, cytokines, and proteins and has long been touted for its medicinal qualities, including natural hydration, high fiber content, laxative and diuretic effect, antiaging impact, antimicrobial properties, and energy enhancement. [6] Saat et al. compared rehydration after exercise with coconut water, carbohydrate-electrolyte beverage and water. [7] They found that coconut water was well tolerated and subjects reported having greater ease in consuming a large amount of coconut water as opposed to an energy drink or water.

Gandhi et al. studied the effect of coconut water consumption on ethylene glycol induced nephrocalcinosis in male Wistar rats. [8] The study demonstrated that coconut water consumption inhibited crystal deposition in renal tissue and decreased the number of crystals in the urine. However, the possible antilithogenic effects of coconut water have never been studied in humans. An anecdotal patient encounter piqued our interest in the potential of coconut water as an antilithogenic natural substance. In the resulting study, our primary goal was to determine the impact of drinking coconut water on known urinary lithogenic factors.

## 2. Materials and Methods

After Institutional Review Board approval, adult volunteers with no prior history of nephrolithiasis were recruited. Each participant was randomized initially to either a coconut water or a water phase. Participants kept meticulous food and fluid intake logs during the first phase of the study and were asked to replicate that diet for the second phase. For each phase, the participant consumed 1.92L of either Taste of Nirvana® pure coconut water or tap water daily for four days. The nutritional facts provided by the manufacturer and percent daily value are shown in Table 1. Participants were not restricted with regard to the consumption of additional fluid of their choice during the study. On days 3 and 4 of each phase the participant collected a 24-hour urine specimen. A washout phase of a minimum of 2 weeks and a maximum of 4 weeks between phases was implemented in this study.

Citrate and malate concentrations of the coconut water were measured using ion chromatography (Dionex, Sunnyvale CA). Electrolytes were measured with ion specific electrodes and pH was measured using a pH electrode. Total alkali of the coconut water was calculated from the citrate and malate concentrations, the beverage pH and the pKs of the anions. The pK of tricarboxylic acid citrate used to calculate anion content was 3.1, 4.7, and 5.4 and for dicarboxylic malate pK was 3.4 and 5.1, respectively. The total alkali is expressed in milliequivalents per liter (mEq/L). Supersaturation levels were calculated using Equil2.

Nonparametric paired analysis using the Wilcoxon test was performed for statistical analysis. Analysis was conducted using SYSTAT v13 (Systat Software, Inc., Chicago IL).

## 3. Results

A total of 8 subjects were recruited into this study: 4 adult males and 4 adult females. The average age of the male participants was 48.5 years (28-69 years) and for female participants 27 years (22-32). Each individual's 24-hour urine collection had a creatinine excretion within 20% of the mean for each subject's four samples corroborating that all samples were collected properly. The two samples from each phase for each individual were averaged. The coconut water itself was also analyzed (Table 2). This showed that the total alkali content was 13.8mEq/L. Each can of Taste of Nirvana® contained 0.48L of coconut water.

The average total urine volume for the participants was 3.03L during both the coconut water and water phase of the studies. Consumption of coconut water significantly increased urinary citrate as compared to tap water by 29% (p=0.02). In addition, consumption of coconut water as compared to tap water increased urinary potassium by 130%(p=0.01) and urinary chloride by 37% (p=0.03) (Table 3). Increases in urinary citrate, potassium, and chloride with consumption of coconut water were similar for males and females and for younger (<=30) and older subjects (>30). Numbers in these subgroups were too small to obtain statistical significance in stratified analyses.

There was no significant alteration in urine volume, urine pH, supersaturation of calcium oxalate and calcium phosphate, urine calcium, and urine sodium.

## 4. Discussion

Hypocitraturia, defined as urinary citrate excretion less than 320mg per day for adults, is an important metabolic abnormality in stone formers with an incidence as high as 63% [9, 10]. Citrate is a well-known inhibitor of calcium stone formation through multiple mechanisms, including complexing with calcium, preventing nucleation of both calcium oxalate and calcium phosphate, and blocking crystal agglomeration and growth [11]. Oral potassium citrate, available in various forms, increases urinary citrate levels and urinary pH; it is the main treatment for hypocitraturia associated nephrolithiasis [12, 13].

Despite its proven efficacy, compliance with potassium citrate therapy is poor. In one study that looked at long-term follow-up of stone formers who were treated with potassium citrate, only 62% consistently took the medication [14]. In addition, given that potassium citrate therapy is costly, upward off $180 USD/month for three times daily dosing of 20 meq, alternative dietary therapies have been evaluated [15]. Lemon juice therapy in the form of lemonade was initially reported to significantly increase urinary citrate levels [16]. Subsequent studies have shown mixed results and have cast some doubt on the effectiveness of lemonade therapy. Koff et al. performed a crossover design trial comparing potassium citrate therapy and lemonade therapy [17]. They found no difference in urinary citrate or urine pH in the lemonade group, while the potassium citrate group demonstrated significant increase in both urinary citrate (20%) and urine pH (8%). Using controlled metabolic conditions, Odvina and colleagues measured urinary stone risk factors and demonstrated that orange juice had a greater alkalinizing and citraturic effect than lemonade [18]; the mean increase in urinary citrate per 240ml of orange juice was 88mg compared to only 11mg during lemonade consumption. Similarly, urinary pH was higher by 0.6 units in the orange juice group compared with lemonade and control phases of the study.

Halebian et al. performed quantitative analysis of citrate content amongst commercially available beverages. Grapefruit juice was found to have the highest citrate content (64.7mmol/L), followed by lemon juice (47.66mmol/L), orange juice (47.36mmol/L), pineapple juice (41.57mmol/L), and home-made lemonade (17.42mmol/L). Crystal Light had the highest concentration of citrate (38.39mmol/L) among non-juice beverages [19]. However, because of how the body absorbs and metabolizes citrate, only a small amount of dietary citrate reaches the urine. Instead, urinary citrate excretion depends closely on acid-base physiologic states. In a state of acid loading, the proximal tubule reabsorbs citrate. On the contrary, during alkali loading, there is decreased renal tubule reabsorption of citrate, which thereby increases urinary citrate excretion [11].

Given the importance of systemic alkalinization and its effect on renal citrate handling, Eisner et al. analyzed lemonade and 15 diet sodas to determine citrate and malate as alkali and the total alkali load. Lemonade had 6.30 mEq/L citrate as alkali, far lower than several other beverages such as Diet-7Up (9.79 mEq/L), Diet Sunkist Orange (8.38 mEq/L), and Sierra Mist Free (8.11 mEq/L). The pH of lemonade is usually less than 3, so most citrate in lemonade is present as citric acid, limiting the amount of alkali delivered. The majority of beverages tested did not have significant measurable malate as alkali, except for Diet Sunkist Orange, Diet Canada Dry Ginger Ale, and Diet Orange Crush. The total alkali content was highest in Diet Sunkist Orange (10.49 mEq/L), Diet-7Up (9.79 mEq/L), and Diet Canada Dry Ginger Ale (8.98 mEq/L) [20]. Of note, coconut water, at 13.8 mEq/L, has far greater alkali content than any of the prior fluids.

In our study, despite relatively low citrate content (2.1 mmol/L), coconut water therapy revealed a significant increase in urinary citrate excretion from baseline (mean increase of 161mg/d). This citraturic effect is likely due to the very high total alkali load (13.8 mEq/L), which is higher than in any of the other juices or nonjuice fluids discussed [20]. The high total alkali load is mainly a function of the high pH of coconut water and the malate content. Of note is that this increase in citrate occurred in nonstone forming individuals with a normal citrate at baseline; whether there would be a similar or greater impact on citrate levels in hypocitraturic stone-formers has yet to be tested. Interestingly, we did not record a significant change in urinary pH. Our findings also revealed significant increase in urinary potassium and chloride, which may be explained by the high potassium and chloride content of coconut water. The coconut water studied contains approximately 1456 mg/L (37.3 mEq) of potassium, which is 31% of the Food and Drug administration daily recommended value for adults [21]. Of note, potassium depletion has been associated with hypocitraturia [11].

Of interest, coconut water contains a significant amount of chloride, which is unusual for a fruit beverage. Potassium content of a beverage is often used as a gauge of alkali content on the assumption that most potassium is accompanied by organic anions. In coconut water, this assumption is not correct as most potassium is actually potassium chloride. This point highlights the need for direct measurement of organic anions and pH to assess the alkali content of a beverage.

The ideal dietary therapy for decreasing urinary stone risk factors should be low in calories, animal protein, sodium, and oxalate and high in citrate and total alkali load. Compared to commercially available grapefruit and orange juice, coconut water has approximately 50% less calories and 60% less sugar content. While there is less overall citrate content compared to other citrus beverages, the very high alkali load is associated with a significant and substantial citraturic effect. Indeed, coconut water may represent a more ideal beverage for increasing urinary citrate compared to lemon juice, lemonade, and other beverages.

To our knowledge, this is the first analysis of coconut water for its antilithogenic properties in humans and the results are promising. Additionally, differences in pH and supersaturation of calcium oxalate between the two groups may have reached significance if sufficiently powered. We chose initially to include only individuals with no prior history of nephrolithiasis to determine if coconut water consumption would change urinary stone risk factors. Future studies with larger sample sizes are needed to evaluate if the citraturic effect of coconut water is operational in calcium stone forming patients with hypocitraturia.

## 5. Conclusions

Coconut water consumption increases urinary potassium, chloride, and citrate in nonstone forming individuals without altering the urine pH.

## Figures and Tables

**Table 1:** Taste of Nirvana coconut water nutritional facts adjusted to 1L serving size and % daily value adjusted according to 2015 FDA guidelines.

	**Nutritional Facts**	%** Daily Value**
**Serving size (L)**	1.0	—
**Calories **	208	—
**Total Fat (g)**	0	0
**Cholesterol (g)**	0	0
**Sodium (mg)**	208	9
**Potassium (mg)**	1456	31
**Total Carbohydrate (g)**	54	41
**Sugar (mg)**	40	—
**Protein (mg)**	0	—
**Chloride**	—	42
**Calcium**	—	13
**Vitamin C**	—	14
**Magnesium**	—	8

**Table 2:** Coconut water analysis. Each can contains 0.48L of coconut water.

	**Taste of Nirvana® Pure Coconut Water**
**pH**	5.41
**Citrate mM/L**	2.1
**Malate mM/L**	5.9
**Total Alkali mEq/L**	13.8
**Na mM/L**	15.7
**K mM/L**	53.0
**Cl mM/L**	53.5

**Table 3:** Mean values from 24-hour urine.

	**Water**	**SD** **∗**	**Coconut Water**	**SD** **∗**	**p-value**
**Volume (L/d)**	3.03	0.6	3.03	0.7	1.00
**SSCaOx**	2.05	0.87	1.82	0.89	0.12
**Ca (mEq/d)**	139	74	137	91	1.00
**Oxalate (mEq/d)**	26.6	9	27.7	7.2	0.40
**Citrate (mEq/d)**	557	207	718	278	0.02
**SSCaP**	0.48	0.3	0.46	0.3	0.78
**pH**	6.3	0.3	6.4	0.3	0.16
**SSUA**	0.31	0.2	0.27	0.2	0.33
**Uric Acid (g/d)**	0.62	0.15	0.614	0.1	0.78
**Na (mEq/d)**	174	33.2	177	46.8	0.89
**K (mEq/d)**	64	14.5	143	30.4	0.01
**Mg (mEq/d)**	97	35.3	101	43	0.58
**P (g/d)**	0.819	0.2	0.82	0.2	0.89
**NH4 (mEq/d)**	36.7	7.8	33.8	9.3	0.33
**Cl (mEq/d)**	174	33.7	238	46.5	0.03
**Sulfate (mEq/d)**	39.6	12.7	42.4	8.6	0.40
**Urea N (g/d)**	10.2	2.5	10.1	2.2	0.67
**Creatinine (mEq/d)**	1588	307	1675	378	0.05

*∗*SD: standard deviation.

## Data Availability

The datasets generated during and/or analyzed during the current study are available from the corresponding author on reasonable request.

## Abbreviations

mEq: Milliequivalents.
